# Incidence and diagnosis of ventilator-associated tracheobronchitis in the intensive care unit: an international online survey

**DOI:** 10.1186/cc13725

**Published:** 2014-02-12

**Authors:** Alejandro Rodríguez, Pedro Póvoa, Saad Nseir, Jorge Salluh, Daniel Curcio, Ignacio Martín-Loeches

**Affiliations:** 1 Hospital Joan XXIII Critical Care Department/IISPV/URV/CIBERES, Mallafre Guasch 4, 43007 Tarragona, Spain; 2 Polyvalent Intensive Care Unit, São Francisco Xavier Hospital, Estrada do Forte do Alto do Duque, 1495-005 Lisboa, Portugal; 3 Pôle de Réanimation, CHU de Lille, 2, avenue Oscar Lambret, 59037 Lille Cedex, France; 4 Institute for Research and Education Postgraduate Program, Instituto Nacional de Câncer Praça Cruz Vermelha, 23 - Centro, 20230-130 Rio de Janeiro, Brazil; 5 Hospital Municipal de Chivilcoy, Av. Hijas de San José 31, 6620 Chivilcoy, Buenos Aires, Argentina; 6 Critical Care Centre, Corporació Sanitària I Universitaria Parc Taulí, Hospital De Sabadell Institut Universitari UAB, Ciber Enfermedades Respiratorias, Parc Taulí, 1 08208 Sabadell, Barcelona, Spain

## Abstract

**Introduction:**

Several aspects of ventilator-associated tracheobronchitis (VAT)—including diagnostic criteria, overlap with ventilator-associated pneumonia (VAP), and appropriate treatment regimens—remain poorly defined. The objectives of this study were to survey reported practices in the clinical and microbiological diagnosis of VAT and to evaluate perceptions of the impact of VAT on patient outcomes.

**Methods:**

We developed a questionnaire consisting of (a) characteristics of the respondent, the ICU, and hospital; (b) current clinical and microbiological diagnostic approach; (c) empirical antibiotic therapy; and (d) the perception of physicians regarding the clinical impact of VAT and its implications.

**Results:**

A total of 288 ICUs from 16 different countries answered the survey: 147 (51%) from the Latin American (LA) group and 141 (49%) from Spain, Portugal, and France (SPF group). The majority of respondents (n = 228; 79.2%) reported making the diagnosis of VAT based on clinical and microbiological criteria, and 40 (13.9%) by clinical criteria alone. Approximately half (50.3%) of the respondents agreed that patients should receive antibiotics for the treatment of VAT. Out of all respondents, 269 (93.4%) assume that a VAT episode increases ICU length of stay, and this perception is greater in the LA group (97.3%) than in the SPF group (89.4%, *P* <0.05). Half of the physicians considered that VAT increases the risk of mortality, and this perception is again greater in the LA group (58.5% versus 41.1%, *P* <0.05).

**Conclusions:**

Given the possible high incidence of VAT and the perception of its importance as a risk factor for VAP and mortality, a large multicenter international prospective study would be helpful to validate a consensual definition of VAT, determine its incidence, and delineate its impact on subsequent VAP occurrence.

## Introduction

Although mechanical ventilation (MV) is potentially life-saving, it also carries significant risks and complications. Of these, ventilator-associated pneumonia (VAP) is one of the most severe, being associated with increased morbidity and duration of MV in the intensive care unit (ICU) [1,2]. Ventilator-associated tracheobronchitis (VAT) is believed to be an intermediate stage between colonization of the lower respiratory tract and VAP. However, more recent data suggests that VAT may be a separate entity that may contribute to increased length of ICU stay and longer duration of MV [3].

Both VAP and VAT are clinically characterized by presence of fever, mucopurulent bronchial secretions, and leukocytosis. In contrast to VAP, VAT does not involve the pulmonary parenchyma and, as a result, does not cause radiographic pulmonary infiltrates. Accurate diagnosis of VAT is challenging, as many conditions commonly encountered in critically ill patients (such purulent secretions, pulmonary edema or acute respiratory distress syndrome) can mimic its signs and symptoms. The current knowledge on VAT, in contrast to that on VAP, is recent and limited to a substantially lower number of large clinical studies. Our main objectives were to document reported practices of clinical and microbiological diagnosis of VAT and to evaluate perceptions of the impact of VAT on patient outcomes. This will serve as a first step of an international prospective study on VAT registered under number NCT01791530 (ClinicalTrials.gov).

## Materials and methods

### Study population

Physicians with a major role in infection control practices and ICU clinical management were surveyed, and only one respondent per ICU was allowed. This study was approved by the ethics committee of Corporació Sanitaria Parc Taulí, Sabadell, Spain (reference number 2013515). Informed consent was not required as the survey consisted of a voluntary anonymous response to a web-based questionnaire, which did not include patient data. The web-based survey attempted to assess the responding physicians’ individual perceptions of current clinical practices.

### Questionnaire

We developed a web-based questionnaire with four parts (Additional file 1): (a) characteristics of the respondent and the ICU and hospital, (b) practices of clinical and microbiological diagnosis of VAT, (c) empirical antibiotic (ATB) therapy used after diagnosis, and (d) the perception of physicians regarding the clinical impact of VAT and the need for treatment. To evaluate diagnostic factors, we questioned investigations performed (for example, fiber-optic bronchoscopy versus less invasive techniques), culture (quantitative or semi-quantitative), and any complementary imaging studies. Regarding ATB use, we included questions on the nature of empirical therapy, combination use, timing of treatment commencement, and the use of inhaled ATB therapy for treatment of VAT. The questionnaire was concise and consisted strictly of multiple-choice questions in an attempt to improve the response rate. It was first developed in Spanish and then translated into English, French, and Portuguese by the Steering Committee members. We transferred the surveys to a web platform (ClinicalRec)—a fine-tuned, hosted web and data-mining platform designed specifically to perform and analyze clinical data—to collect the data.

### Dissemination to target group

The questionnaire was available online from 1 January to 31 March 2013. It was endorsed by SEMICYUC (Sociedad Española de Medicina Intensiva, Crítica y Unidades Coronaria), FEPIMCTI (Federación Panamericana e Ibérica de Sociedades de Medicina Crítica y Terapia Intensiva), and BRICNET (Brazilian Research in Intensive Care NETWORK). The members of the Steering Committee (local opinion leaders) were responsible for the distribution of the survey in their countries. The questionnaire was sent by e-mail to 1,036 physicians: 610 (58.8%) in Spain (n = 350), Portugal (n = 50), and France (n = 210), which formed the Spain, Portugal, and France (SPF) group, and 426 in the Latin American (LA) group. Table 1 shows the number of surveys sent by country. The survey was not distributed in the US, although one participant ICU which was part of a research network actively collaborating with different centers in Spain was included.

**Table 1:** Survey response rate

**Country**	**Total of sent surveys, n (%)**	**Responses, n (%)**
Spain	350 (33.8)	92 (26.3)
France	210 (20.3)	32 (15.2)
Portugal	50 (4.8)	17 (34.0)
Brazil	182 (17.6)	61 (33.5)
Colombia	60 (5.8)	22 (38.6)
Argentina	50 (4.8)	17 (34.0)
Chile	30 (2.9)	12 (40.0)
Ecuador	20 (1.9)	12 (60.0)
Peru	20 (1.9)	7 (35.0)
Mexico	20 (1.9)	1 (5.0)
Venezuela	10 (0.9)	4 (40.0)
Uruguay	10 (0.9)	4 (40.0)
Bolivia	10 (0.9)	4 (40.0)
Guatemala	8 (0.8)	2 (25.0)
Costa Rica	5 (0.5)	1 (20.0)
USA	1 (0.1)	1 (100)

### Statistical analysis

Descriptive statistics were used to characterize the study sample. We used chi-square and Mann-Whitney tests to compare survey characteristics of study participants and to make comparisons by country and region. Data were processed by using the Statistical Package of Social Sciences (SPSS) 13.0.1 standard version (IBM, Chicago, IL, USA). Response rates and sample characteristics were analyzed by using descriptive statistics. In descriptive data analysis, proportions (percentages) were reported. Statistical significance was defined as a *P* value of less than 0.05.

## Results

**Figure 1:**
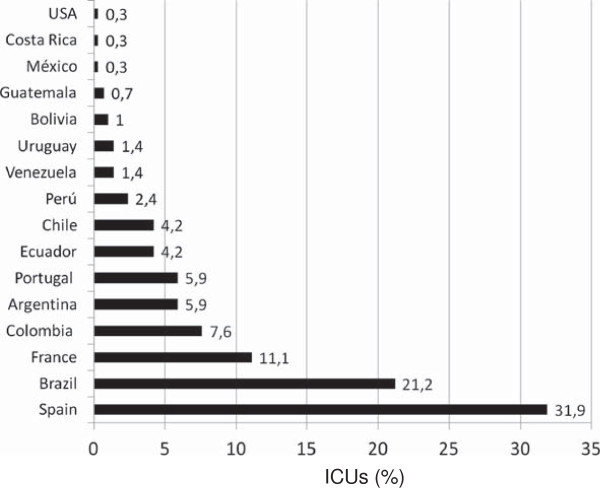
**Percentage of intensive care units (ICUs) that responded to the survey out of a total of 288 responses.** Data are presented according to the country of origin.

A total of 288 ICUs from 16 different countries replied to the survey, representing a response rate of 27.8% (288/1,036). Fifty-one percent (n = 147) were from the LA group, and 141 (49%) from the SPF group (Figure 1). The number of individual responses per country is shown in Table 1. The main characteristics of the respondents and the ICUs were compared and are also presented in Table 2. In the SPF group as compared with LA, more hospitals were in a public health system, had greater numbers of beds, and were more frequently university-associated. However, the only ICUs with more than 50 beds were reported in the LA group. There was no significant statistical difference when comparing the response of physicians according to the type of ICU (university versus non-university ICU), nor when comparing the number of ICU beds (less or more than 20).

**Table 2:** Characteristics of the respondents and intensive care unit setting

**Characteristics**	**Global**	**SPF group**	**Latin American group**	***P *** **value**^**a**^
	**n = 288**	**n = 141**	**n = 147**	
	**n (%)**	**n (%)**	**n (%)**	
Number of beds in ICU				
>50 beds	11 (3.8)	-	11 (7.5)	0.003
21-50 beds	79 (27.4)	40 (28.4)	39 (26.5)	0.82
10-20 beds	140 (48.6)	80 (56.7)	60 (40.8)	0.01
<10 beds	58 (20.1)	21 (14.9)	37 (25.2)	0.04
ICU type				
General	278 (96.5)	137 (97.2)	141 (95.9)	0.79
Cardiac surgery	3 (1.0)	-	3 (2.0)	0.26
Neurotrauma	2 (0.7)	-	2 (1.4)	0.49
Surgical	2 (0.7)	2 (1.4)	-	0.46
Respiratory	2 (0.7)	1 (0.7)	1 (0.7)	1.00
Trauma	1 (0.3)	1 (0.7)	-	0.98
Number of beds in hospital				
>500 beds	103 (35.8)	79 (56.0)	24 (16.3)	<0.001
201-500 beds	107 (37.2)	55 (39.0)	52 (35.4)	0.60
100-200 beds	50 (17.4)	7 (5.0)	43 (29.3)	<0.001
<100 beds	28 (9.7)	-	28 (19.0)	<0.001
Hospital type				
Public	188 (65.3)	133 (94.3)	55 (37.4)	<0.001
Private	78 (27.1)	5 (3.5)	73 (49.7)	<0.001
Mixed	22 (7.6)	3 (2.1)	19 (12.9)	0.002
Academic degree				
University	190 (66.0)	104 (73.8)	86 (58.5)	0.009
No university	98 (34.0)	37 (26.2)	61 (41.5)	0.009

^a^All comparisons were made between Spain, Portugal, and France (SPF) and Latin American groups. ICU, intensive care unit.

### Diagnosis of ventilator-associated tracheobronchitis

Almost all (99.7%) of the respondents considered that ventilated patients are at risk of developing VAT. One hundred two respondents (35.5%) considered that VAT is more frequent in all ICU ventilated patients irrespective of the type of admission diagnosis. Eighty-nine (30.9%) respondents considered that patients admitted because of medical conditions are at higher risk of VAT, followed by neurological (n = 53; 8.4%) and surgical (n = 20; 7.0%) patients.

The majority of respondents (n = 228; 79.2%) make the diagnosis of VAT on the basis of both clinical and microbiological criteria. Forty (13.9%) reported using clinical criteria alone, and 19 (6.6%) consider it as a diagnosis of exclusion. Endotracheal aspirates were reported to be the most frequently used technique for the diagnosis of VAT (59.4%), followed by bronchoalveolar lavage (BAL) (13.9%) and multiple sampling techniques (12.2%). BAL and mini-BAL techniques were more frequently employed by the LA group, whereas multiple sample techniques were more commonly used by the SPF group (Table 3), and 47.6% (n = 137) used any bronchoscopic technique for diagnosis of VAT (Table 4). Whereas the use of bronchoscopic techniques is part of routine practice in only 3.1% of respondents, it is used in 35.8% when the chest x-ray (CXR) is not conclusive and in 8.7% when ATB treatment is started. Similarly, more than 50% of physicians requested a computed tomography (CT) scan to confirm or exclude the diagnosis of VAT, 2.4% as routine practice, and 48.3% when CXR is inconclusive (Table 4).

**Table 3:** Techniques used for the diagnosis of ventilator-associated tracheobronchitis

**Technique**	**Global**	**SPF group**	**Latin American group**	***P *** **value**^**a**^	**OR (95% CI)**^**a**^
	**n = 288**	**n = 141**	**n = 147**		
	**n (%)**	**n (%)**	**n (%)**		
Endotracheal aspirate (ETA)	171 (59.4)	87 (61.7)	84 (57.1)	0.50	ND
Bronchoalveolar lavage (BAL)	40 (13.9)	12 (8.5)	28 (19.0)^a^	0.01	0.4 (0.2-0.8)
ETA plus other techniques	35 (12.2)	27 (19.1)	8 (5.4)^a^	0.001	4.1 (1.7-10.3)
Mini-BAL	21 (7.3)	4 (2.8)^a^	17 (11.6)^a^	0.009	0.2 (0.07-0.08)
Protected specimen brush	4 (1.4)	3 (2.1)	1 (0.7)	0.58	ND
No response	17 (5.9)	8 (5.7)	9 (6.1)	1.00	ND

^a^All comparisons were made between Spain, Portugal, and France (SPF) and Latin American groups. CI, confidence interval; ND, not determined; OR, odds ratio.

**Table 4:** Microbiological techniques and complementary studies used to the diagnosis of ventilator-associated tracheobronchitis

**Questions**	**Global**	**SPF group**	**Latin American group**	***P *** **value**	**OR (95% CI)**
	**n = 288**	**n = 141**	**n = 147**		
	**n (%)**	**n (%)**	**n (%)**		
Quantitative cultures					
Yes	217 (75.3)	97 (68.8)^a^	120 (81.6)	0.01	0.5 (0.3-0.9)
No	66 (22.9)	42 (29.8)^a^	24 (16.4)	0.01	2.2 (1.9-3.9)
NR	5 (1.7)	2 (1.4)	3 (2.0)	1.00	ND
Gram stain technique					
Yes	116 (40.3)	62 (44.0)	54 (36.7)	0.25	ND
No	170 (59.0)	78 (55.3)	92 (62.6)	0.25	ND
NR	2 (0.7)	1 (0.7)	1 (0.7)	1.00	ND
Bronchoscopy for the diagnosis of VAT					
Never	151 (52.4)	73 (51.8)	78 (53.1)	0.92	ND
Only when the chest x-ray is not conclusive	103 (35.8)	50 (35.5)	53 (36.1)	1.00	ND
Only if I decide to start ATBs	25 (8.7)	13 (9.2)	12 (8.2)	0.91	ND
Always	9 (3.1)	5 (3.5)	4 (2.7)	0.95	ND
CT scan for the diagnosis of VAT					
Never	142 (49.2)	82 (58.2)^a^	60 (40.8)	0.005	2.0 (1.2-3.3)
Only when the chest x-ray is not conclusive	139 (48.3)	58 (41.1)^a^	81 (55.1)	0.02	0.5 (0.3-0.9)
Always	7 (2.4)	1 (0.7)	6 (4.1)	0.14	ND

^a^All comparisons were made between Spain, Portugal, and France (SPF) and Latin American groups. ATB, antibiotic; CI, confidence interval; CT, computed tomography; ND, not determined; NR, no response; OR, odds ratio; VAT, ventilator-associated tracheobronchitis.

Although the majority of respondents (n = 276; 95.8%) use microbiological findings to guide ATB treatment, more than half (n = 170; 59.0%) do not perform a Gram stain on the respiratory sample. Sixty-six (22.9%) reported that they do not request quantitative cultures of respiratory secretions as routine practice, and this occurred more frequently in the SPF group (Table 4).

### Treatment of ventilator-associated tracheobronchitis

Approximately half (50.3%) of the respondents agreed that patients diagnosed with VAT should receive ATBs. Only 24.3% (n = 70) routinely prescribed ATBs, whereas 42.0% (n = 121) only prescribed ATBs in the presence of hemodynamic instability. Conversely, 26% (n = 75) of physicians considered that VAT should not be treated with ATB, and 7.6% (n = 22) answered that they do not have a clear opinion on the most appropriate treatment. A total of 90.3% (n = 260) of the responders indicated that the duration of MV helped in the decision of ATB treatment; however, only 50% of them indicated this option in the survey in regard to the best treatment option for VAT (Table 5).

**Table 5:** Antibiotic treatment of ventilator-associated tracheobronchitis

**Questions**	**Global**	**SPF group**	**Latin American group**	***P *** **value**^**a**^	**OR (95% CI)**^**a**^
	**n = 288**	**n = 141**	**n = 147**		
	**n (%)**	**n (%)**	**n (%)**		
All VAT patients should receive ATB treatment?					
Yes	121 (42.0)	59 (41.8)	62 (42.2)	1.00	ND
No	75 (26.0)	31 (22.0)	44 (29.9)	0.16	ND
Only in patients with cardiovascular failure	70 (24.3)	41 (29.1)	29 (19.7)	0.08	1.6 (0.9-2.9)
Unknown	22 (7.6)	10 (7.1)	12 (8.2)	0.90	
Which is the most appropriate treatment for VAT?					
Broad-spectrum IV ATBs	84 (29.2)	37 (26.2)	47 (32.0)	0.34	ND
Narrow-spectrum IV ATBs	20 (6.9)	15 (10.6)	5 (3.4)	0.002	3.3 (1.1-10.9)
Select ATBs according to MV days	145 (50.3)	74 (52.5)	71 (48.3)	0.55	ND
Nebulized ATBs	6 (2.1)	2 (1.4)	4 (2.7)	0.71	ND
Broad-spectrum IV ATBs + nebulized ATBs	7 (2.4)	1 (0.7)	6 (4.1)	0.14	ND
Never	26 (9.0)	12 (8.5)	14 (9.5)	0.92	ND
Which is the most appropriate option for treatment of VAT?					
IV ATBs in monotherapy	180 (62.5)	99 (70.2)	81 (55.1)	0.01	1.9 (1.1-3.2)
IV ATBs in combination	60 (20.8)	20 (20.6)	31 (21.1)	0.16	ND
IV ATBs + nebulized ATBs in monotherapy	27 (9.4)	7 (5.0)	20 (13.6)	0.02	0.3 (0.1-0.8)
IV ATBs + nebulized ATBs in combination	16 (5.6)	2 (1.4)	14 (9.5)	0.006	0.1 (0.02-0.6)
No reply Timing to start ATB treatment?	5 (1.7)	4 (2.8)	1 (0.7)	0.34	ND
<12 hours	211 (73.3)	96 (68.1)	115 (78.2)	0.07	0.5 (0.3-1.0)
13-24 hours	44 (15.3)	23 (16.3)	21 (14.3)	0.75	ND
>24 hours	24 (8.3)	17 (12.1)	7 (4.8)	0.04	2.7 (1.01-7.5)
Never ATB treatment	9 (3.1)	5 (3.5)	4 (2.7)	0.95	ND
ATB duration					
7-10 days	25 (8.7)	7 (5.0)	18 (12.2)	0.04	0.3 (0.1-0.9)
7-10 day but de-escalation	167 (58.0)	84 (59.0)	83 (56.5)	0.67	ND
14 days	1 (0.3)	0	1 (0.7)	1.00	ND
Until clinical resolution	22 (7.6)	13 (9.2)	9 (6.1)	0.44	ND
<7 days	71 (24.7)	35 (24.8)	36 (24.5)	1.00	ND
No reply	2 (0.7)	2 (1.4)	0	0.90	ND

^a^All comparisons were made between Spain, Portugal, and France (SPF) and Latin American groups. ATB, antibiotic; CI, confidence interval; IV, intravenous; MV, mechanical ventilation; ND, not determined; NR, no response; OR, odds ratio; VAT, ventilator-associated tracheobronchitis.

**Figure 2:**
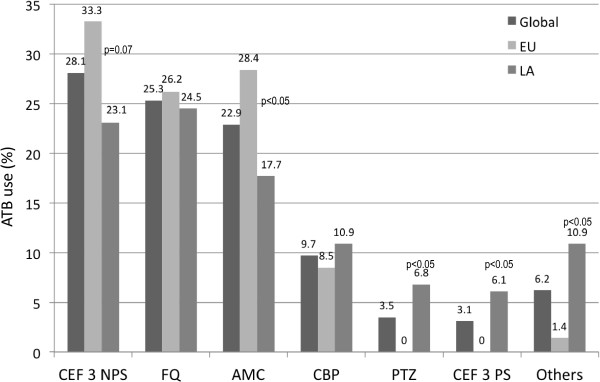
**Empiric antibiotic therapy for early ventilator-associated tracheobronchitis.** AMC, amoxicillin/clavulanate; AMK, Amikacin; CBP, carbapenem; CEF3 NPS, third-generation non-Pseudomonal cephalosporins; CEF3 PS, third-generation Pseudomonal cephalosporins; EU, European Union group; FQ, fluoroquinolones; LA, Latin American group; PTZ, Piperacillin/Tazobactam; Vanco: vancomycin.

**Figure 3:**
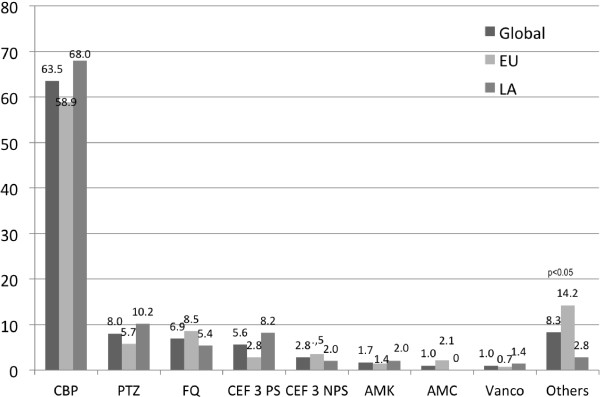
**Empiric antibiotic therapy for late ventilator-associated tracheobronchitis.** AMC, amoxicillin/clavulanate; AMK, Amikacin; CBP, carbapenem; CEF3 NPS, third-generation non-Pseudomonal cephalosporins; CEF3 PS, third-generation Pseudomonal cephalosporins; EU, European Union group; FQ, fluoroquinolones; LA, Latin American group; PTZ, Piperacillin/Tazobactam; Vanco: vancomycin.

Intravenous (IV) monotherapy (62.5%) is the first choice of ATB treatment for VAT and was more frequently supported by the SPF group, followed by IV ATB combination (20.8%) and then IV and nebulized ATB. The use of nebulized ATB is more frequently reported by the LA group compared with the SPF group (Table 5).The majority of respondents (73.3%) started ATB treatment within 12 hours of VAT diagnosis. Initiation of treatment after 24 hours of VAT diagnosis was more commonly reported by the SPF group (12.1%) as compared with the LA group (4.8%). More than half (66.7) indicated they favored ATB treatment with a duration of between 7 and 10 days. Half of the respondents preferred to de-escalate therapy when the results of microbiology tests are available. Surprisingly, only 24% indicated a preference for a short course of ATB treatment (<7 days). The empiric antimicrobial regimens for early- and late-onset VAT are shown in Figures 2 and 3, respectively.

### Impact of ventilator-associated tracheobronchitis

Finally, 94.1% (n = 274) of respondents believe that the development of VAT is associated with longer duration of MV. Two hundred sixty-nine (93.4%) assume that a VAT episode is associated with increased ICU length of stay, and this perception is greater in the LA group (97.3%) as compared with the SPF group (89.4%, *P* <0.05). Half of the physicians considered that VAT increases the risk of mortality, and this perception is again greater in the LA group as compared with the SPF group (58.5% versus 41.1%, *P* <0.05).

## Discussion

This is the first international survey that has aimed to evaluate perceptions of impact and self-reported practices in the diagnosis and treatment of VAT. We consider that the present survey is relevant to increase the current knowledge of VAT. There are several conflicting results regarding the clinical implications of VAT, and the present survey provides a path toward meaningful research questions for future clinical studies. The main conclusions of the present study are that VAT is perceived as a common complication of MV in ICU patients and is a diagnosis based not only on clinical criteria but also on non-invasive techniques and microbiological confirmation and that half of physicians surveyed use systemic ATBs to treat VAT as they believe that VAT is associated with a longer duration of MV and longer ICU length of stay.

The true incidence of VAT is not currently known. VAT is recognized by the Centers of Disease Control and Prevention (CDC)/National Healthcare Safety Network [4] as an individual clinical entity. As the clinical relevance of VAT seems to be increasing, it is necessary to further delineate its incidence in a study that applies a widely accepted definition. A large multicenter international study is planned in order to answer some of the questions presented in this survey. The goal of the present survey was to determine the importance of VAT and physicians attitudes based on the answers taken from 288 ICUs in Europe and Latin America.

The majority of respondents (>90%) to the present survey agreed that patients under MV have a significant risk of developing VAT, and they perceived that this risk was even higher in medical patients. This finding is in opposition to that of the study by Malacarne and colleagues [5], who in a prospective epidemiological study conducted in 71 Italian ICUs with 9,493 consecutive patients found that surgical patients were more likely to develop VAT than medical patients (odds ratio (OR) of 1.64). According to the current definition, as recently published by the CDC, a diagnosis of VAT is made if there is absence of pneumonia in the x-ray and at least two of the following findings: (1) fever (>38°C), (2) cough, (3) new or increased production of sputum, (4) rhonchi, and (5) wheezing, and at least one of the following: (a) positive culture obtained by deep tracheal aspirate or bronchoscopy and (b) positive laboratory test on respiratory secretions [4].

Similarly, the European Respiratory Society, European Society of Clinical Microbiology and Infectious Diseases, and European Society of Intensive Care Medicine taskforce requires a positive culture of respiratory secretions as a mandatory item in the diagnosis of VAT [6]. In the present survey, almost 80% of physicians routinely diagnosed VAT with the assistance of microbiological studies, and a minority (13.9%) relied solely on clinical assessment. As expected, non-invasive techniques were more frequently preferred to obtain samples and achieve microbiological confirmation. Although the use of quantitative cultures of respiratory secretions could be helpful to differentiate VAT from colonization, invasive techniques are not mandatory for VAT diagnosis. It was therefore surprising that invasive techniques are used by almost half of respondents. This finding is more frequent in the LA group than the SPF group.

The subjectivity and variability inherent in interpretation of CXRs in mechanically ventilated patients make chest imaging ill suited for inclusion in a definition algorithm to be used for the potential purposes of public reporting, inter-facility comparisons, and pay-for-reporting and pay-for-performance programs. Several authors [7,8] have proposed the use of CT lung scans and this recommendation is followed by half of the respondents to the survey. Nevertheless, it is important to consider whether a CT scan for this purpose is cost-effective and safe for critically ill patients. Ventilated patients are at high risk for complications en route, and in addition transport outside the ICU has been reported to be an independent risk factor for VAP (OR 2.9, 95% confidence interval 1.4 to 5.7) [8,9]. A recent observational cross-sectional study of adult patients in the emergency department found that CXRs demonstrated poor sensitivity and positive predictive value for detecting pulmonary opacities when compared with chest CT for routine clinical care [10,11]. It remains unclear whether the use of chest CT is cost-effective or even necessary for the management of suspected VAT.

The ATB treatment of VAT remains controversial [12]. In the survey, half of the respondents reported that the duration of MV is an important factor to consider when deciding on administration of ATBs to patients with VAT. The use of ATBs in VAT has been evaluated in two recent randomized controlled trials. Palmer and colleagues [13] found faster weaning and less use of systemic ATB when nebulized ATBs were administered. Nseir and colleagues [14] found a lower mortality rate and more MV-free days. Although the administration of aerosolized ATB in patients with uncomplicated VAT is an attractive approach, it is reported by only the minority of the respondents. In our survey, this practice is more frequent in LA. Well-designed prospective studies are needed in order to further delineate the best therapeutic approach for suspected VAT.

We acknowledge limitations to the present survey. First, the present study may have a bias selection as VAT may represent an important clinical entity for respondents. Second, although we sought to survey the ICU physician most involved in the decision-making process related to ICU infections, it may represent the personal opinion of the respondents and may not reflect hospital or country-wide policies. Third, we could not be completely sure that respondents were not using VAT and VAP answers in an interchangeable fashion. However, questions about VAT were formulated with closed answers, and questions related to VAP were made in order to avoid confusion and misinterpretation between these two entities. Fourth, we recognize that self-reported practices in a survey may not reflect actual practice of the respondents, a limitation inherent in the nature of all surveys. Finally, this survey comes from only three European and 13 LA countries and thus cannot represent the opinions of these entire communities.

## Conclusions

VAT is recognized as a frequent complication of MV. However, subjective components within VAT definition and diagnosis may impact the reliability and accuracy of case identification. VAT represents a clinical entity that is closer to the clinical reality than more strict criteria usually considered in most clinical published studies. Given the perceived increasing incidence of VAT and its importance as a possible risk factor for VAP and other adverse outcomes, a large multicenter international prospective study, with standardized clinical and microbiologic criteria for VAT diagnosis, needs to be performed. This should aim to validate an accepted definition of VAT, determine its incidence, and further delineate its impact on clinically relevant outcomes.

## Key messages

• Ventilator-associated tracheobronchitis is perceived as a frequent complication of mechanical ventilation.

• The diagnosis of ventilator-associated tracheobronchitis is usually based on a combination of clinical and microbiological criteria.

• More than half of respondents did not perform a Gram stain on the respiratory sample and one out of four did not request a sample for quantitative culture in the diagnosis of ventilator-associated tracheobronchitis.

• Half of physicians reported prescribing broad-spectrum systemic antibiotics for the treatment of ventilator-associated tracheobronchitis.

• The majority of physicians believed that ventilator-associated tracheobronchitis is associated with a longer duration of mechanical ventilation and longer intensive care unit length of stay.

## Abbreviations

ATB: antibiotic; BAL: bronchoalveolar lavage; CDC: Centers of Disease Control and Prevention; CT: computed tomography; CXR: chest x-ray; ICU: intensive care unit; IV: intravenous; LA: Latin American; MV: mechanical ventilation; OR: odds ratio; SPF: Spain, Portugal, and France; VAP: ventilator-associated pneumonia; VAT: ventilator-associated tracheobronchitis.

## Competing interests

The authors declare that they have no competing interests. The study was endorsed by SEMICYUC (Sociedad Española de Medicina Intensiva, Crítica y Unidades Coronaria) and FEPIMCTI (Federación Panamericana e Ibérica de Sociedades de Medicina Crítica y Terapia Intensiva). The endorsing societies had no role in study design; collection, analysis, or interpretation of data; writing of the manuscript; or the decision to submit the manuscript for publication. The content is solely the responsibility of the authors and does not necessarily represent the official views of the societies.

## Authors’ contributions

IM-L assisted in the design of the study, coordinated patient recruitment, analyzed and interpreted the data, and helped to make substantial contributions to the concept, design, analysis, and interpretation of data. He assisted in writing the manuscript, was involved in revising it critically for important intellectual content, revised the final version, and acted as guarantor of/person responsible for the entire manuscript. The TAVeM group made important contributions to the acquisition and analysis of data. AR assisted in the design of the study, coordinated patient recruitment, analyzed and interpreted the data, and helped to make substantial contributions to the concept, design, analysis, and interpretation of data. He assisted in writing the manuscript and revised the final version. PP, JS, SN, and DC assisted in the design of the study, coordinated patient recruitment, analyzed and interpreted the data, and assisted in writing the manuscript and were involved in revising it critically for important intellectual content. All authors read and approved the final manuscript.

## Supplementary Material

Additional file 1: **Survey submitted for the TAVeM group.** Web-based questionnaire for the ventilator-associated tracheobronchitis (VAT) in the Intensive Care Unit International Online Survey. Click here for file
