# Supplementary material for: Incidence and diagnosis of ventilator-associated tracheobronchitis in the intensive care unit: an international online survey
Source: Crit Care. 2014 Feb 12;18(1):R32. doi: 10.1186/cc13725 (PMC4057509; doi:10.1186/cc13725)
Supplement: Additional file 1: — Survey submitted for the TAVeM group. Web-based questionnaire for the ventilator-associated tracheobronchitis (VAT) in the Intensive Care Unit International Online Survey. [file cc13725-S1.pdf]

# APPENDIX 1

## GTEI/SEMICYUC SURVEY
## VENTILATOR-ASSOCIATED TRACHEOBRONCHITIS (VAT)

| | |
|---|---|
| **Country** | **City** |
| **Nº of total beds at the Hospital**<br>a) <100<br>b)100-200<br>c) 201-500<br>d) >500 | **Nº of ICU beds**<br>a) <10<br>b) 10-20<br>c) 21-50<br>d) > 50 |
| **Type of Hospital**<br>a) Public<br>b) Private<br>c) Mixed<br><br>**Academic degree of the Hospital**<br>a) University<br>b) Non-university | **Type of ICU**<br>a) Medical /mixed<br>b) Neurologic<br>c) General surgery<br>d)Cardiovascular surgery<br>e)Burns<br>f)Medical cardiovascular<br>g)Medical respiratory |
| **1.- In what type of patients do you consider VAT more frequently?**<br>a) All the ICU patients<br>b)Medical patients<br>c)Surgical patients<br>d)Neurological patients<br>e)Trauma patients<br>f) Unstable patients<br>g) Unstable patients (independently of the cause)<br>h) COPD patients | **2.- Which are the criteria that you usually use for diagnosis of VAT?**<br>a) Only clinical criteria (fever + purulent respiratory secretion)<br>b) Clinical criteria plus microbiological criteria<br>c) by exclusion |
| **3.- Which are the microbiologic methods that you usually use for the diagnosis of VAT?**<br>a) Gram stain of respiratory secretions<br>b) Qualitative culture of respiratory secretions<br>c)Quantitative culture of respiratory secretions ($10^5$ ETA; $10^4$ BAL; $10^3$ PSB) | **4.- Which diagnostic technique commonly used for the diagnosis of VAT?**<br>a) Tracheal aspirate<br>b)Broncho-alveolar lavage (BAL)<br>c) Mini-BAL<br>d)Protected brush (PSB) |
| **5.- In your opinion, which are the situations to perform a bronchoscopy for the diagnosis of VAT?**<br>a) Never<br>b)Always<br>c) Only if I decide to start an antibiotic treatment<br>d) Only when the chest x-ray is not conclusive | **6.- In your opinion, which are the situations to perform a CT scan for the diagnosis of VAT?**<br>a) Never<br>b) Always<br>c) Only when the chest x-ray is not conclusive |
| **7.- Do you consider that the microbiological documentation is useful to select the appropriate antibiotic treatment?**<br>a)Yes<br>b) No | **8.- If you receive a negative Gram stain of respiratory secretion from patients with VAT suspected; do you wait for the culture results before start the antibiotic treatment?**<br>a) Yes<br>b) No |
| **9.- Which is your antimicrobial strategy to manage a patients with VAT in the ICU?**<br>a) None<br>b) Always start with broad-spectrum intravenous (IV) antibiotic (AB)<br>c) Always start with narrow-spectrum IV AB<br>d)Select the AB according the mechanical ventilation (MV) days<br>e)Always start with nebulized AB<br>f) Always start with broad-spectrum IV + nebulized AB | **10.- Do you consider that all the patients with VAT must receive antibiotic treatment?**<br>a) Yes<br>b) No<br>c) I don't know<br>d) Only in patients with cardiovascular and/or respiratory failure |
| **11.- In which type of patients with VAT do you consider to indicate antibiotic treatment?**<br>a) All the ICU patients<br>b) Medical patients<br>c) Surgical patients<br>d) Neurological patients<br>e) Trauma patients<br>f) Unstable patients (independently of the cause)<br>g) COPD patients | **12.- When do you usually start the antibiotic treatment in patients with VAT?**<br>a) <12 h<br>b) 12-24 h<br>c) 25-48 h<br>d) > 48h<br>e) Never |
| **13.- In your opinion; which is the most appropriate option for treating patients with VAT?**<br>a) intravenous (IV) antibiotic (AB) in monotherapy<br>b) IV AB in combination<br>c) IV AB plus nebulized AB in monotherapy<br>d) IV AB plus nebulized AB in combination | **14.- If you decide to start and antibiotic (AB) treatment for VAT ….**<br>a) Do you maintain the initial AB treatment for 7-10 days?<br>b) Do you maintain AB 7 -10 days but de-escalate when receive the microbiologic results?<br>c) Do you maintain AB 14 days?<br>d) Do you maintain AB until the resolution of the clinical parameters (fever + respiratory secretions)?<br>e)Do you use short AB treatment (< 7days) |
| **15.- Do you decide the selection of the antibiotic (AB) treatment according to the characteristics of the VAT (early or late)?**<br>a) Yes<br>b) No | **16.- Do you consider that VAT increases the mechanical ventilator days?**<br>a) Yes<br>b) No |
| **17.- Do you consider that the VAT increases the ICU length of stays?**<br>a) Yes<br>b) No | **18.- Do you consider that the VAT increases the ICU patient's mortality?**<br>a) Yes<br>b) No |
| **19.- Which antibiotic/s do you usually use for treating patients with early-onset VAT** | **20.- Which antibiotic/s do you usually use for treating patients with late-** |

| | |
|---|---|
| **(< 5 day of mechanical ventilation)?**<br>a) Carbapenems<br>b) Fluoroquinolones<br>c) Third generation non-Pseudomonal cephalosporins<br>d) Amoxicillin-clavulanate<br>e) Amikacin<br>f) Polymixuns<br>g) Vancomicyn<br>h) Linezolid<br>i) others | **onset VAT (> o =5 day of mechanical ventilation)?**<br>a) Carbapenems<br>b) Fluoroquinolones<br>c) Third generation Pseudomonal cephalosporins<br>d) Amoxicillin-clavulanate<br>e) Amikacin<br>f) Polymixuns<br>g) Vancomicyn<br>h) Linezolid<br>i) others |
| **21.- Are the physicians of your ICU familiar with the use of nebulized antibiotic for treating patients with VAT?**<br>a) Yes<br>b) No | **22.- Do you consider the use of nebulized antibiotics useful for treating patients with VAT?**<br>a) Yes<br>b) No |
| **23.- Which antibiotic do you use to nebulizer in patients with VAT?**<br>a) Colistin<br>b) Tobramicyn<br>c) Amikacin<br>d) others | |
